# *Drosophila* as a model for the gut microbiome

**DOI:** 10.1371/journal.ppat.1008398

**Published:** 2020-04-23

**Authors:** William B. Ludington, William W. Ja

**Affiliations:** 1 Department of Embryology, Carnegie Institution for Science, Baltimore, Maryland, United States of America; 2 Department of Biology, Johns Hopkins University, Baltimore, Maryland, United States of America; 3 Department of Neuroscience, The Scripps Research Institute, Jupiter, Florida, United States of America; 4 Center on Aging, The Scripps Research Institute, Jupiter, Florida, United States of America; University of Massachusetts, Worcester, UNITED STATES

## Introduction

The microbiome has tremendous potential to impact host physiology and metabolism [1]. Gut bacteria in particular have been linked to diverse functions and specific diseases [2]. Mechanistic studies remain challenging in part due to the complexity of the mammalian gut microbiome, which can vary greatly between individuals and is composed of approximately 1,000 species of microorganisms [3]. Invertebrate systems are fruitful models for dissecting complex host–microbe interactions. In particular, *Drosophila melanogaster*, the fruit fly, is one of the most powerful models for animal genetics and has a simple microbiome composed of 5 to 20 microbial species that can be reconstituted in the lab by brief treatment of eggs with bleach followed by association with defined bacterial species [4, 5]. Thus, the fly model facilitates exploration of both host and bacterial genetics.

Fly-associated microbes have been studied for over a century [6, 7], and recent publications have described how host immune effectors and bacterial genes influence the composition of the microbiome [8–11]. Bacterial presence impacts various fly phenotypes including development [12], behavior [13, 14], life span [15], and disease resistance [16]. Since ingestion of microorganisms contributes to a substantial portion of the macronutrient and micronutrient intake of flies, it is unclear to what extent the effects of the microbiome are due to resident gut microbes versus microbes serving as agricultural goods for consumption from the food surface [17–20]. In the lab, gut microbe composition is highly variable, and these microbes can be lost if flies do not continue to ingest live bacteria [21, 22]. However, recent work has identified gut symbionts that stably colonize specific niches in the gut [23, 24]. We propose that identifying these symbionts is a critical step in establishing a fly model for the gut microbiome.

### Bacteria can stably colonize the fly gut

**Fig 1:**
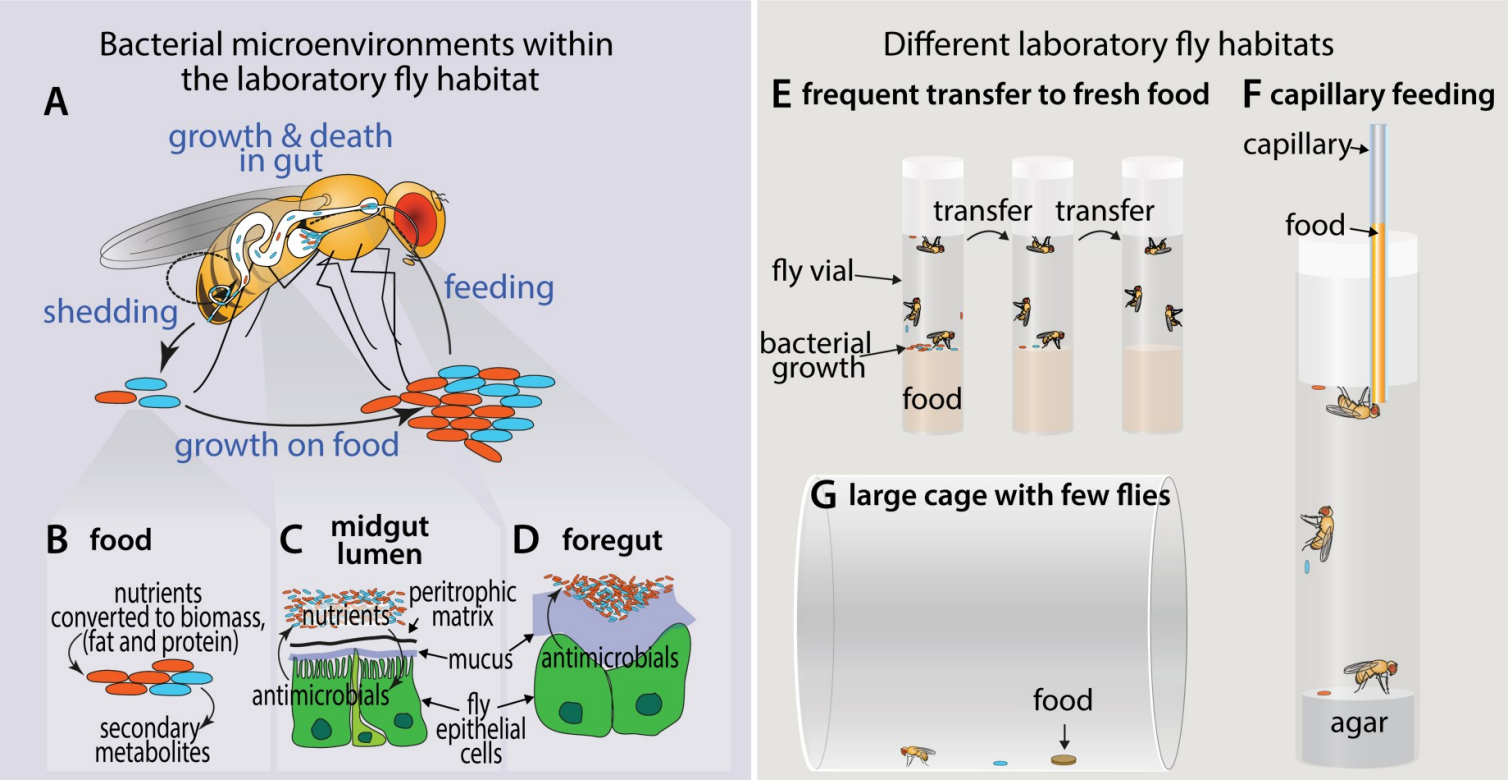
A microbe’s perspective on the laboratory fly. (**A**) In the laboratory fly vial, many bacteria grow on the food, where they are consumed by both larval and adult flies. A smaller population of bacteria are harbored in the fly gut, where they can be actively consumed by the fly or shed back onto the food. Processes on the food and in the fly gut select for different bacterial compositions [25]. (**B**) Bacterial metabolism on the food changes the composition of the provided media, converting sugars and other carbohydrates to more protein-rich and fat-rich bacterial biomass, which can contribute to fly nutrition [17–20]. (**C**) Digestive processes in the fly midgut kill bacteria, liberating their nutrients for absorption by the intestinal epithelial cells. Fly cells are protected from the bacteria by a peritrophic matrix [26]. (**D**) Large populations of bacteria can stably occupy the crop and cardia in the fly foregut [23, 24]. (**E–G**) Stable versus transient colonization of the fly gut can be distinguished by minimizing exposure to environmental bacteria. These techniques include (**E**) frequent transfers to fresh food [18, 22], (**F**) capillary feeding [24], and (**G**) housing low fly numbers in large enclosures [23].

In the lab, flies live on top of their food, which serves as a rich substrate for microbial growth (**Fig 1A–1D**). Recent studies have attempted to decouple the influence of fly food bacteria and fly gut bacteria by (1) frequent transfer to fresh, sterile food [18, 22]; (2) use of large enclosures with low numbers of flies [23]; or (3) capillary feeding, whereby bacteria have no substrate for growth outside of the gut [24] (**Fig 1E–1G**). These techniques reveal that different bacteria occupy different microenvironments within the fly and its enclosure [25].

Obadia and colleagues [24] took advantage of the capillary-feeding approach to quantify colonization of individual flies after ingesting a single dose of a bacterial strain isolated from wild flies, lab flies, or humans. They found that colonization is probabilistic, with the odds increasing with higher doses. A spectrum of colonization stability was identified between different bacterial strains that largely depended upon their origin—bacterial strains from wild flies tended to stably colonize even at low doses. Out of the 17 strains screened, 11 were categorized as stable colonizers, including the genera *Lactobacillus*, *Leuconostoc*, *Enterococcus*, and *Acetobacter*.

Using an alternative approach to identify bona fide gut colonizers (**Fig 1G**), Pais and colleagues [23] showed that *Acetobacter thailandicus* stably colonizes the gut of *D*. *melanogaster* but not the gut of the closely related *D*. *simulans*. Thus far, stable colonizers appear to favor regions such as the foregut (crop and proventriculus [also known as cardia]), suggesting that, as in mammals, mechanisms exist to colonize specific niches [23, 24]. Colonization is limited to certain fly species [23] or bacterial strains [24], suggestive of underlying mechanisms of specificity. Both host and bacterial mechanisms likely drive this specificity. For example, on the host side, the acidic copper cell region of the midgut is known to limit bacterial survival [27]. On the bacterial side, Winans and colleagues [28] used a metagenome-wide association approach to examine the evolutionary signature of selection on laboratory-fly–associated bacteria. The study revealed that genes for flagellar motility were lost and genes for tolerance of nitrogenous waste were acquired—potentially the consequence of relaxed selective forces on homogeneous food in the lab and frequent cycling of bacteria between gut and external habitats. Future studies might use similar approaches on validated gut colonizers to determine whether these genes and others impact host–microbe interactions that lead to stable colonization. Strategies that the fly host and resident microbes use to form stable associations may be informative on how the mammalian gut microbiome develops.

### Fly–microbe symbiosis

Pais and colleagues [23] further discovered a mutual growth enhancement in *A*. *thailandicus* and its *D*. *melanogaster* host that was not conferred to *D*. *simulans*, implying an evolved symbiosis. Storelli and colleagues [29] found similar evidence for an evolved mutualism with *Lactobacillus plantarum*. A feeding or foraging preference for specific bacteria was found independently by several labs [13, 30, 31]. Thus, fly-associated bacteria can colonize the host, benefit the host, and benefit from the host, and the host seeks them out. Together, these features make *Drosophila* a strong model system to study the mechanisms of symbiotic associations between animals and their bacteria. Whether resident gut bacteria are required for the various organismal phenotypes is still unclear, given the difficulty in separating the impacts of food and gut bacteria in many of these studies. However, the combined body of evidence suggests an evolved mutualistic relationship between flies and their bacteria that has specific mechanisms at molecular, cellular, organismal, and ecological scales. While a variety of models will be necessary to unravel the complexities of the microbiome, these features of *Drosophila* suggest it can be an integral part of the overall mission to understand host-microbiome systems.

### The same bacterial abundance can be achieved through different rates of growth and death

Microbiome studies typically quantify the relative abundance of different bacterial species, providing snapshots of a dynamic gut microbial community that do not take into consideration the total abundance of bacteria in the gut or the turnover of cells due to bacterial growth, death, and shedding. Recently developed methods to measure the turnover of bacterial cells inside the fly gut uncovered high-turnover rates [24] and spatial structure [32]. Different stably associated strains of the same bacterial species, *L*. *plantarum*, undergo widely different turnover rates [24]. One consequence of this is that the fly receives a very different nutritional contribution from these different strains despite a similar bacterial abundance. Thus, similar strains with similar abundance in the gut can have very different nutritional impacts on the host. The techniques developed in flies for quantifying microbial growth and death rates may be generalized to microbiome dynamics in mammalian models.

### *Drosophila* can model mammalian gut complexity

Microbiome impacts on the host may result from direct effects of the individual bacterial species or from interactions between them. Interactions between *Lactobacillus* and *Acetobacter* species affect fly fat content [33], and genes involved in lactate and acetoin metabolism underlie the bacterial mutualism [34]. The ease of generating gnotobiotic flies and modularity of the fly-microbiome facilitates combinatorial studies to explore the impacts of complexity. Combinatorial experimental designs have shown that higher-order interactions between 3, 4, and 5 species of the microbiome affect fly life history strategy [35, 36]. Interactions between commensals and pathogens also influence host health. *Vibrio cholera* strains modulate their type VI secretion system depending on which commensal bacterial strains are present, and this determines whether *Vibrio* kills the fly [37]. Despite the tractability of the fly-microbiome model, identifying underlying mechanisms driving organismal phenotypes will be difficult to decouple from nutritional influences. Small changes in microbial growth rates, both in the gut or in the habitat, can vastly change nutritional contributions to the fly. Testing whether supplementation with heat-killed bacteria phenocopies live bacterial inoculations might help untangle some of these effects, but studies rarely provide biomass equivalent to live bacterial growth. We speculate that the strength of the fly model in uncovering mechanisms of host–microbe interactions is augmented by the effect of the microbiome on organismal phenotypes.

## Conclusion

The simple microbiome of flies and ease of rearing gnotobiotic animals makes them an attractive model to incorporate into studies of the gut microbiome. Recent discovery of stable colonizing strains, the development of techniques to isolate the gut microbiome from bacterial growth on the food, and the ability to quantify turnover of the population of gut bacterial cells allow unprecedented tractability in this powerful genetic model animal.
